# Greater Trochanteric Pain Syndrome and the Efficacy of Platelet-Rich Plasma Injections: A Systematic Review

**DOI:** 10.7759/cureus.72597

**Published:** 2024-10-29

**Authors:** Hamza Ahmed, Muhammad Yasir Tarar, Aizaz Khalid, Numan Shah, Aima Gilani, Maham Ijaz

**Affiliations:** 1 Trauma and Orthopaedics, Salford Royal NHS Foundation Trust, Manchester, GBR; 2 Trauma and Orthopaedics, Wythenshawe Hospital, Manchester, GBR; 3 General Surgery, University Hospitals Sussex NHS Foundation Trust, Chichester, GBR; 4 Neurosurgery, Salford Royal NHS Foundation Trust, Manchester, GBR

**Keywords:** greater trochanteric pain syndrome, gtps, lateral hip pain, platelet rich plasma, prp injections

## Abstract

Greater trochanteric pain syndrome (GTPS) is one of the most prevalent causes of lateral hip pain. The incidence rate is as high as 1.8 patients per 1000 annually, with females predominantly affected. We compared and analysed the effectiveness of platelet-rich plasma (PRP) injections in treating GTPS.

Literature search was carried out on PubMed, Embase and Cochrane by two independent reviewers using the terms: ‘Greater Trochanteric Pain syndrome’ and ‘Platelet-rich plasma'. The Preferred Reporting Items for Systematic Reviews and Meta-Analyses (PRISMA) guidelines were followed, and the Cochrane risk of bias tool and Methodological Index for Non-Randomized Studies (MINORS) tool were used to assess bias. Nine studies were shortlisted and reviewed for patient sample size, diagnostic modalities, the presence of tendinopathy or bursitis, the number of PRP injections administered, and the length of symptom relief achieved.

We analysed nine studies between 2013 to 2024 comprising of a total of 508 patients who received treatment with PRP injections for lateral hip pain. There was an improvement and sustained relief in symptoms in eight studies, while one reported no change. Many studies indicated PRP injections to be more effective than corticosteroid injections (CSI) in treating GTPS.

PRP appears to be an effective injectable treatment option for GTPS, which does not respond to conservative therapy. However, due to the limitations of the current literature, there is a need for more large-scale, high-quality randomized clinical trials to assess further the effectiveness of PRP for treating GTPS.

## Introduction and background

Greater trochanteric pain syndrome (GTPS) is a prevalent cause of lateral hip pain in acute orthopaedic settings [1,2]. The pain is aggravated by walking, climbing stairs, standing or sitting on the affected leg for a long period of time, crossing your legs and rigorous physical activity. It was formerly known as trochanteric bursitis, but radiological and histological studies have shown that it mainly involves tendinopathy of the gluteus medius and minimus [1], with or without associated bursitis. There is no established treatment protocol, and approaches range from conservative to surgical management [3].

GTPS normally does tend to settle with conservative management options like anti-inflammatory medications or having adequate rest; however, in severe cases, there may be the need for more invasive options such as surgery, corticosteroid injections, and platelet-rich plasma (PRP) injections. In recent times, the use of platelet-rich plasma injections is becoming increasingly popular in the field of orthopaedics as it is known to enhance tissue healing in a minimally invasive way by delivering platelet-derived growth factors to the affected region. 

This review aims to compare and analyse the effectiveness of available treatment options, focusing primarily on platelet-rich plasma (PRP) injections. It will also compare it with other treatment modalities at our disposal such as corticosteroid injections. 

## Review

Materials and methods

We assessed the studies reporting the use of PRP injections in GTPS. Literature search of three online databases was carried out on PubMed (1978 to present), Embase (1974 to present) and Cochrane (1988 to present). It was conducted by two independent reviewers using the terms: ‘Greater Trochanteric Pain syndrome’ and ‘Platelet-rich plasma’. Duplicates were removed, yielding 246 articles. Any article that reported the efficacy of platelet-rich plasma in the management of greater trochanteric pain syndrome was included. Reviews, meta-analysis and abstracts were excluded from our analysis. All full texts were retrieved. The systematic review was carried out using Preferred Reporting Items for Systematic Reviews and Meta-Analyses (PRISMA) guidelines [4]. The Cochrane risk of bias tool and Methodological Index for Non-Randomized Studies (MINORS) tool was used to assess bias. Nine studies were then shortlisted for review.

**Figure 1:**
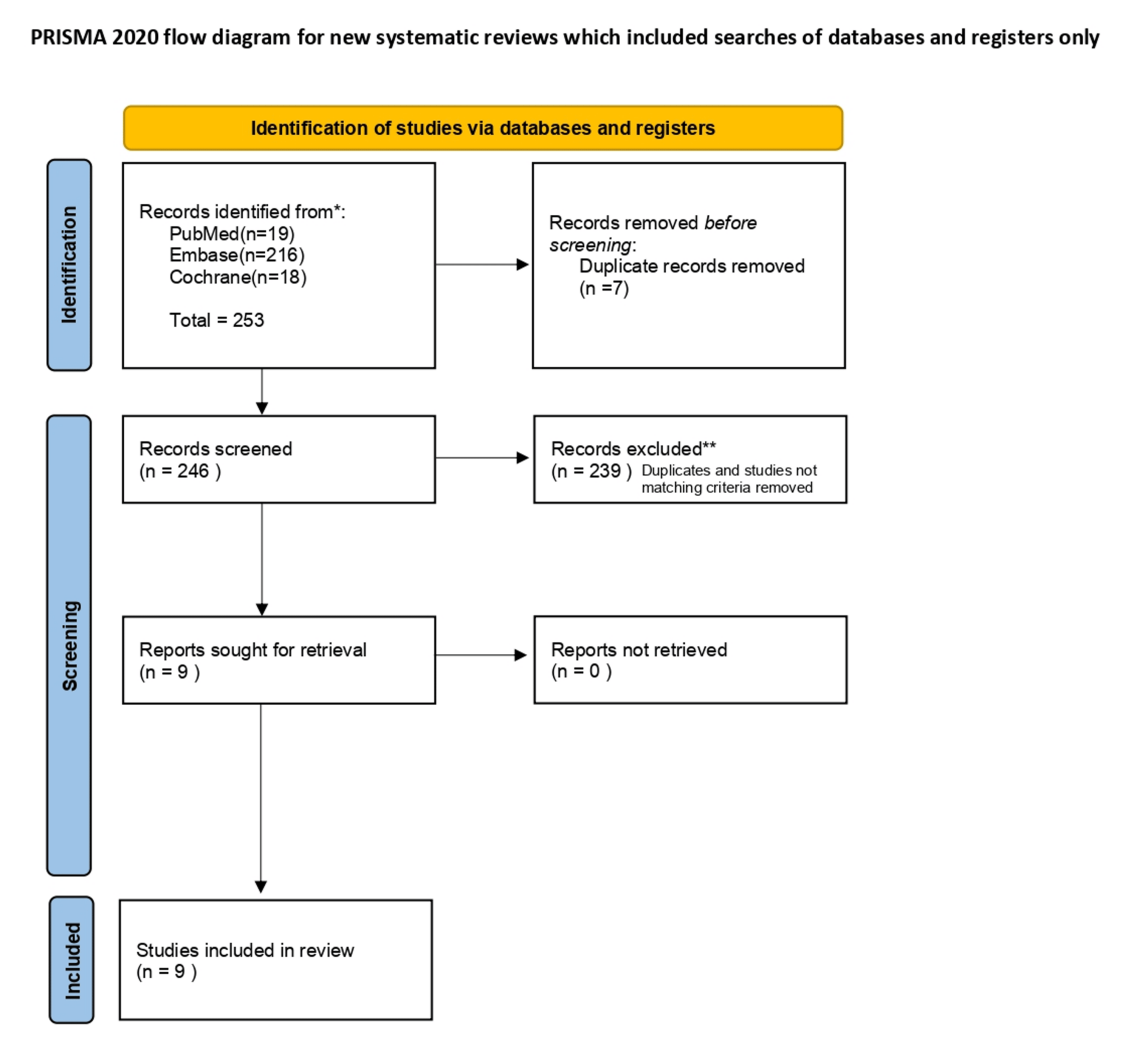
PRISMA diagram PRISMA: Preferred Reporting Items for Systematic Reviews and Meta-Analyses

We evaluated the patient sample size, diagnostic modalities, the presence of tendinopathy or bursitis, the number of PRP injections administered, and the length of symptom relief achieved.

Results

**Table 1:** Nine studies shortlisted reporting the use of PRP injections for GTPS PRP: Platelet-rich plasma; GTPS: greater trochanteric pain syndrome; HIPPO: Hip Injections PRP vs Placebo Trial.

Study name	Year	Number of patients	Tendinopathy (Medius and Minimus)	Bursitis	Diagnosis is made using USG/MRI	Number of PRP injection(s)	Duration of relief
Greater Trochanteric Pain Syndrome: Percutaneous Tendon Fenestration Versus Platelet-Rich Plasma Injection for Treatment of Gluteal Tendinosis [5]	2016	30	Yes	No	Yes	Unknown	Improvement at 13 weeks
Ultrasound-guided Platelet-rich Plasma Application Versus Corticosteroid Injections for the Treatment of Greater Trochanteric Pain Syndrome: A Prospective Controlled Randomized Comparative Clinical Study [6]	2020	24	Yes	N/A	Yes	Unknown	Improvement at 24 weeks
Leucocyte-Rich Platelet-Rich Plasma Treatment of Gluteus Medius and Minimus Tendinopathy: A Double-Blind Randomized Controlled Trial With 2-Year Follow-up [7]	2019	80	Yes	No	N/A	Single	Improvement at 2 years
The Effectiveness of Platelet-Rich Plasma Injections in Gluteal Tendinopathy: A Randomized, Double-Blind Controlled Trial Comparing a Single Platelet-Rich Plasma Injection With a Single Corticosteroid Injection [8]	2018	80	Yes	No	Yes	Single	Improvement at 12 weeks
Platelet-rich Plasma Treatment in Patients with Osteoarthritis of the Hip and Greater Trochanteric Pain Syndrome [9]	2018	42	Yes	N/A	Yes	Three	Improvement at 24 weeks
Ultrasound-Guided Subfascial Platelet-Rich Plasma Injections Versus Enthesis Needling for Greater Trochanteric Pain Syndrome: A Randomized Controlled Trial [10]	2024	92	Yes	No	Yes	Unknown	Improvement at 12 weeks
Ultrasound-Guided Platelet Rich Plasma (PRP) Injections for Greater Trochanteric Pain Syndrome (GTPS): A Retrospective Case Series [11]	2013	10	Yes	No	Yes	Unknown	Improvement at 12 weeks
A Double-Blind Randomised Control Trial Investigating the Efficacy of Platelet-rich Plasma versus Placebo for the Treatment of Greater Trochanteric Pain Syndrome (the HIPPO Trial): A Protocol for a Randomised Clinical Trial [12]	2018	102	Yes	N/A	N/A	Unknown	Improvement at 12 weeks
No Attributable Effects of PRP on Greater Trochanteric Pain Syndrome [13]	2019	48 (24 PRP and 24 control)	Yes	No	Yes	Single	At 12 weeks – no improvement noted

Jacobson et al. (2016) compared ultrasound-guided percutaneous tendon fenestration with PRP injections for the treatment of GTPS [5]. The study included 30 patients and found a 79% improvement in symptoms after 92 days.

Begkas et al. (2020) conducted a randomized comparative clinical study [6] to evaluate PRP therapy against corticosteroid injections (CSI) for treating GTPS, analysing 24 patients. The study found that PRP therapy yielded superior clinical outcomes at 24-week follow-up.

Similarly, Fitzpatrick et al. (2019) conducted a double-blinded, randomized controlled trial with a two-year follow-up [7], building on prior research indicating that patients with chronic gluteal tendinopathy experienced greater clinical improvement at 12 weeks with a single platelet-rich plasma (PRP) injection compared to those receiving a single corticosteroid injection. The study found that LR-PRP injections provide benefits for up to two years, while corticosteroid injections offer maximum relief for up to six weeks and not beyond 24 weeks. The trial included 80 patients, with a mean age of 60 years and a female-to-male ratio of 9:1.

Fitzpatrick et al. (2018) carried out a randomized controlled trial [8] involving 80 patients with an average age of 60 years and a female-to-male ratio of 9:1. The study found that patients suffering from chronic gluteal tendinopathy for over four months experienced greater clinical improvement after 12 weeks when treated with a single PRP injection compared to those who received a single corticosteroid injection.

Shirokava et al. (2018) conducted a comparative analysis [9] to evaluate the clinical effectiveness of two treatment approaches: a course of three periarticular injections of platelet-rich plasma (PRP) administered weekly, versus a single injection of a glucocorticosteroid drug (GCS). Out of the 71 patients selected, 42 underwent PRP therapy. The PRP therapy reduced pain intensity, as measured by the Visual Analog Scale (VAS), for up to six months, whereas the effects of GCS therapy lasted only three months.

Atilano et al. (2024) analysed 92 patients to evaluate the clinical effectiveness of subfascial platelet-rich plasma (PRP) injections versus enthesis needling for treating greater trochanteric pain syndrome [10]. Most participants (90%) were women with an average age of 55 years. The PRP group showed significantly greater improvement from baseline to 12 months after treatment, with 66% of patients reporting a reduction in pain at 12 weeks.

A retrospective case series conducted by Massimi et al. (2013) assessed the effectiveness of PRP injections for GTPS [11] in 10 patients aged 30 to 85 years, with nine of them being female. MRI scans were used to diagnose tendinosis or partial tears. Approximately 80% of the patients reported improvement when evaluated at around 12 weeks.

Oderuth et al. (2018) set up a Hip Injections PRP vs Placebo Trial (HIPPO) study design [12] to test the hypothesis that PRP injections are effective in treating GTPS in patients who have a failed experience with conservative management. The study is a randomized controlled trial (RCT), which is double-blinded and conducted at a single centre. It aims to compare the clinical effectiveness of PRP with a placebo injection of normal saline. The trial will conclude after the final patient completes their 12-month follow-up and is still ongoing.

All the studies have a few common factors among them. Each study demonstrated improved clinical outcomes for patients after receiving PRP injections. There was tendinopathy addressed in all of them. The majority of the patients in these studies were female and the average age was greater than 50 years. In addition to this, patients were selected at random. The minimum presentation duration for a patient was three months. Lastly, most diagnosis for GTPS was based primarily on radiological evidence.

Debatable Outcomes

Even though recent evidence suggests that PRP injections are highly effective in improving clinical outcomes for GTPS, there is still some controversial data. Thompson and Pearson (2019) assessed whether a single PRP injection could decrease pain intensity in individuals with GTPS [13]. Participants with chronic lateral hip pain were randomly assigned to receive either a PRP injection (24 people) or a saline injection (24 people), with both groups being prescribed the same unconventional exercise regimen. No differences in any outcomes were observed between the two groups at any of the follow-up points.

Discussion 

Epidemiology of GTPS 

The incidence rate of GTPS is as high as 1.8 patients per 1000 annually [1]. It predominantly affects women [14], with a significant increase in cases during the fourth to sixth decades of life [15]. Females generally have a wider pelvis relative to their overall body width compared to males. This results in more prominent trochanters, which subsequently increases the tension of the iliotibial band over them. Additionally, the lower femoral neck shaft angle causes greater compression of the gluteus medius muscle on the greater trochanter, potentially leading to lateral hip pain.

Causes of GTPS

Chronic lower back pain is a significant cause of GTPS [16]. Other causes include hip trauma or surgery [17], excessive or repetitive exercise leading to friction [3], prolonged sitting or standing [18], weakened gluteal muscles [19], hip osteoarthritis [2], having a sedentary lifestyle [20], and differences in pelvic shape [21]. Any of these factors can contribute to increased stress and repetitive friction between the greater trochanter and iliotibial band, leading to microtrauma of the gluteal tendons attaching to the greater trochanter resulting in pain.

Investigations

A precise medical history and examination are often sufficient for diagnosing GTPS [22]. The physical examination includes the jump sign [1] and the single leg stance test [23], which are highly sensitive and have a strong positive predictive value for confirming MRI findings related to GTPS [24]. Additional tests include flexion, abduction and external rotation (FABER) test, adduction test (ADD) test, flexion, adduction and external rotation (FADER) test, positive Trendelenburg test, positive Ober’s test and a positive step-up and -down test. 

When the clinical diagnosis is uncertain, imaging techniques such as ultrasound and MRI are very helpful [24]. Ultrasound is particularly effective with a high positive predictive value (PPV) and can reveal tears in the gluteus medius or minimus tendons [24], inflammatory changes, or a fluid-filled thickened trochanteric bursa.

Treatment Modalities

The management of GTPS includes both conservative and surgical approaches [3,19]. Conservative management strategies may involve a variety of options, either alone or in combination, such as non-steroidal anti-inflammatory drugs [25], physiotherapy and rehabilitation [26], local corticosteroid injections [27], platelet-rich plasma (PRP) injections, and low-energy extracorporeal shockwave therapy [28].

If conservative treatment is insufficient to alleviate symptoms for the patient or the situation becomes more severe, surgery might be required. The surgical options for managing GTPS include gluteal tendon repair [29], trochanteric reduction osteotomy [30], iliotibial band release [31], and bursectomy [32].

Role of PRP in GTPS

*Composition and working*: Platelet-rich plasma consists of plasma, the liquid part of blood that is about 90% water, along with a high concentration of platelets [33], which is approximately three to five times greater than usual. A blood sample is collected from the patient and subjected to centrifugation, which separates the blood components and concentrates the platelets in the plasma.

This is then injected into the targeted area, such as a tendon or injured knee. The activated platelets release cytokines and growth factors [34] that encourage tissue regeneration and support cell reproduction, which can lead to faster healing, reduced pain, and even stimulate hair growth.

The use of local anaesthetics in these procedures is debated as they can significantly impair platelet function [35], though they do not affect the release of growth factors. Further research is needed to clarify their impact.

Different Types of PRP Injections

As PRP injections become increasingly popular, various formulations have emerged. These include leucocyte-rich PRP, which contains platelets and white blood cells, Leucocyte-poor PRP primarily contains platelets with a few white blood cells, and autologous conditioned serum contains growth factors derived from white blood cells without whole cells. In addition to these, there is a high demand for platelet lysate as well that contains growth factors derived from platelets without whole cells.

Current research suggests that each formulation is effective for specific conditions. For example, leucocyte-ich PRP has shown benefits in treating GTPS [7] and other tendinopathies due to its superior ability to stimulate tenocyte proliferation [36]. In contrast, leucocyte-poor PRP is more advantageous for joint sprains, ligament tears, and osteoarthritis [37].

However, these studies have certain limitations, and more extensive research is needed to determine the most effective formulation for each condition.

Timing

There isn’t a strict guideline for the best time to administer PRP, but it is generally recommended around three months after symptoms appear [38] when the condition is identified as chronic.

Single vs Multiple Injections

The effectiveness of multiple PRP injections for pain relief varies across different studies. A single PRP injection lowers the pain score, but multiple PRP injections tend to lessen pain severity [39] in the subsequent three months. However, there is no difference in pain at six months.

Role of PRP in Similar Other Conditions

PRP injections are employed for a variety of conditions, ranging from musculoskeletal pain and injuries to cosmetic applications. These injections can be effective for treating numerous musculoskeletal issues, such as chronic tendon injuries [40] like tennis elbow [41] or jumper’s knee. PRP is also sometimes used for persistent neck and back pain [42].

Early research suggests that PRP injections might alleviate pain and stiffness associated with osteoarthritis [43] by modifying the joint environment and reducing inflammation. Additionally, PRP injections can benefit certain rotator cuff injuries [44].

In the realm of hair restoration, PRP injections are utilized to treat male pattern baldness [45] by preventing hair loss and encouraging new hair growth. In plastic surgeries, especially face lift procedures, it can cause decreased oedema of the face [46]. It has also reduced the risk of sternal wound infections [47] following cardiac surgery.

Contraindications

PRP injections have specific contraindications [48], including conditions such as blood disorders, low platelet counts, sepsis, anaemia, and cancer. Hence, it is important to know the past medical and surgical history of the patient ideally before proposing this treatment option to them. 

## Conclusions

PRP appears to be a promising, safe and effective injectable treatment option for GTPS that does not respond to conservative therapy. This review has demonstrated sustained relief in the symptoms of GTPS on using PRP over a period of time. However, due to the limitations of the current studies, there is a need for large-scale, high-quality randomized clinical trials to further assess the effectiveness of PRP for treating GTPS and establish definitive results.
